# Gender-Specific Associations of Serum Antibody to *Porphyromonas gingivalis* and Inflammatory Markers

**DOI:** 10.1155/2015/897971

**Published:** 2015-01-31

**Authors:** Michiko Furuta, Yoshihiro Shimazaki, Shunichi Tanaka, Kenji Takeuchi, Yukie Shibata, Toru Takeshita, Fusanori Nishimura, Yoshihisa Yamashita

**Affiliations:** ^1^Section of Preventive and Public Health Dentistry, Division of Oral Health, Growth and Development, Kyushu University Faculty of Dental Science, Fukuoka 812-8582, Japan; ^2^Department of Preventive Dentistry and Dental Public Health, School of Dentistry, Aichi-Gakuin University, Nagoya 464-8650, Japan; ^3^Japanese Red Cross Kumamoto Healthcare Center, Kumamoto 861-8528, Japan; ^4^Section of Periodontology, Division of Oral Rehabilitation, Kyushu University Faculty of Dental Science, Fukuoka 812-8582, Japan

## Abstract

It remains unclear whether serum antibody titer against *Porphyromonas gingivalis* (*Pg*) and inflammatory components lead to periodontal deterioration in each gender, as periodontal and systemic status is influenced by gender. The present study investigates the gender-specific probable effects of titer against *Pg* and inflammatory markers on periodontal health status in a longitudinal study. A retrospective study design was used. At two time points over an 8-year period (in 2003 and 2011), 411 individuals (295 males with a mean age of 57.6 ± 11.2 years and 116 females with a mean age of 59.2 ± 10.3 years) were surveyed. Periodontal status, serum antibody titer against *Pg*, and high-sensitive C-reactive protein (hsCRP) were evaluated. Poisson regression analyses revealed that the elevated titer against *Pg* and hsCRP significantly predicted the persistence of periodontal disease 8 years later in females with periodontal disease in 2003. Elevated hsCRP was significantly associated with the incidence of periodontal disease 8 years later in females who were periodontally healthy in 2003. Males had a weaker association among titer against *Pg*, inflammatory markers, and periodontal disease. These findings suggest that immune response to *Pg* infection in addition to inflammatory components affects periodontal deterioration in females.

## 1. Introduction

Oral diseases limit an individual's capacity in biting, chewing, smiling, speaking, and psychosocial wellbeing [1]. The most common oral diseases are dental caries and periodontal disease. Periodontal disease, that is, inflammatory disorder of the gingiva, is highly prevalent around the world, and nearly 90% of adults have periodontal disease [2].

As with many chronic diseases, periodontal disease has multiple risk factors, and it is important to treat both the local and systemic factors [3]. Among local factors for periodontal disease, it has been recognized that periodontal disease is caused by specific bacteria in the periodontal pocket [4].* Porphyromonas gingivalis *(*Pg*) plays a major role in the pathogenesis of periodontal disease and is considered to induce elevated systemic and local immune responses in periodontal patients [5, 6]. Elevated serum IgG antibody levels of* Pg* have previously been reported to be closely connected to the presence of* Pg* in periodontal pockets [7, 8], reflecting the notion that serum antibody titers against* Pg* are higher in periodontal patients than in healthy individuals [9–11]. Most previous studies have been cross-sectional or short-term longitudinal in design and such designs do not provide information or are deficient in information on the long-term association between serum antibody titers against* Pg* and periodontal status.

Periodontal disease is a local inflammatory condition and is linked to systemic inflammation via host responses. Several cross-sectional studies have reported that levels of inflammatory markers are higher in patients with periodontal disease than in healthy individuals [12, 13]. Systemic inflammation accompanies chronic inflammatory diseases such as cardiovascular disease, diabetes, and metabolic syndrome [14], and thus systemic inflammation is suggested to be an underlying risk factor in periodontal disease as a localized inflammatory disease.

As another risk factor for periodontal disease, gender is an important consideration, because periodontal disease is often reported to be more prevalent or severe in males than in females [3, 15, 16] and associations between periodontal disease and metabolic syndrome have been confirmed in females but not in males [16]. Thus, the association between periodontal disease and local and systemic risk factors may also be expected to have gender differences. However, most studies have not considered this hypothesis and did not use stratified analysis, which would allow determination of whether the relationship is unique to one gender, or even opposite in males and females. In this study, we investigated the gender-specific probable effects of titer against* Pg* and inflammatory markers on periodontal status in a longitudinal study, as it remains unclear whether these factors lead to gender-specific periodontal deterioration.

## 2. Materials and Methods

### 2.1. Study Participants

**Figure 1:**
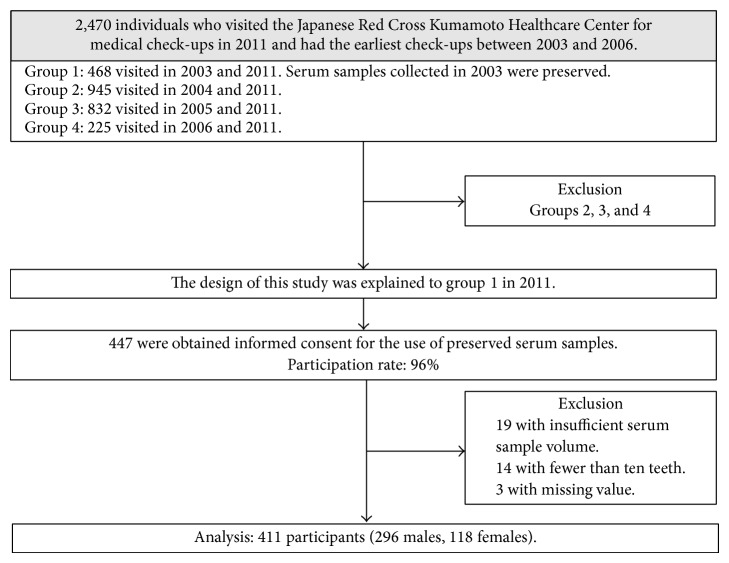
Flow diagram of study participant selection.

We performed a retrospective study. Participants were recruited from among 2,470 individuals who visited the Japanese Red Cross Kumamoto Healthcare Center, Kumamoto, Japan, for periodic medical check-ups including dental examination in 2011 and had the earliest check-ups between 2003 and 2006. The 2,470 individuals were categorized into four groups: 468 visited in 2003 and 2011 (group 1), 945 visited in 2004 and 2011 (group 2), 832 visited in 2005 and 2011 (group 3), and 225 visited in 2006 and 2011 (group 4). In group 1, serum samples collected in 2003 for another study had been preserved. The design of this study using preserved serum samples for measurement of titers against* Pg *and high-sensitive C-reactive protein (hsCRP) was explained to 468 individuals (group 1) and written informed consent was obtained from 447 individuals (participation rate, 96%). Nineteen participants with insufficient serum sample volume were excluded. We also excluded 17 participants with missing value and fewer than 10 teeth due to difficulties in assessing their current periodontal health properly [16]. Therefore, 411 participants (295 males with a mean age of 57.6 ± 11.2 years and 116 females with a mean age of 59.2 ± 10.3 years) were analyzed in this study. Participant flow diagram is presented in Figure 1.

The study was approved by Kyushu University Institutional Review Board for Clinical Research (22-71).

### 2.2. Oral and General Examinations and Questionnaire

One dentist (ST) assessed the oral health status of participants in both 2003 and 2011. The number of teeth present was determined. According to the World Health Organization Community Periodontal Index (CPI) criteria [17] with modification, periodontal condition was assessed in all present teeth to monitor the periodontal health. The highest CPI codes were recorded in each sextant. Periodontal disease was defined as at least one sextant with the presence of periodontal pocket depth ≥4 mm (CPI code ≥ 3) [18].

Overweight was defined as body mass index (BMI) of 25.0 or greater. A venous blood sample was drawn and analyzed for fasting glucose, triglycerides, high density lipoprotein (HDL) cholesterol, leukocyte count, and differential leukocyte count (i.e., neutrophils, acidocytes, and monocytes). Elevated levels of fasting glucose, triglycerides, HDL, and blood pressure were defined by the Joint Interim Societies [19]. Elevated leukocytes were defined as white blood cell of ≥9.0 × 10^9^/L [20]. In the present study, we measured hsCRP in preserved serum samples in 2003. HsCRP levels of ≥1.0 mg/L were defined as elevated hsCRP [21]. CRP levels tend to increase with acute bacterial and viral infections, age, smoking, myocardial infarction, rheumatoid arthritis, obesity, type 2 diabetes, hypertension, and cancer [22]. We considered the effect of these factors on hsCRP in analysis.

Information on smoking habit, alcohol consumption, and toothbrushing frequency was obtained by a self-administered questionnaire. Smokers were categorized as never smokers, who had never smoked regularly; past smokers, who had smoked regularly but had stopped smoking more than one year ago; and current smokers, who had smoked regularly. Alcohol drinkers were categorized as either current drinker, who had drunk at least one time per week, or not current drinkers. Toothbrushing frequency was categorized as either more than three times daily toothbrushing or not.

### 2.3. Measurement of Titers against Pg

The immunological assays used in this study were as described previously [11, 23]. Serum IgG antibody titers against* Pg* (FDC381) were determined by Leisure Inc. (Tokyo, Japan) using enzyme-linked immunosorbent assay (ELISA) from serum samples stored at –20°C. The absorbance of each sample was evaluated and assigned ELISA unit (EU) values relative to the absorbance of a pool of sera collected from periodontally healthy control individuals [11].* Pg* antibody levels are expressed as standardized values calculated as follows: (EU for study serum samples – EU for control samples)/2 × (SD of control samples) [11]. An elevated serum antibody titer against* Pg* was defined as having a value greater than median value [24].

### 2.4. Statistical Analysis

Chi-squared test for categorical data and Mann-Whitney* U* test for continuous data were used to determine significant differences (*P* < 0.05, two sided) between males and females or to elevate the associations between periodontal disease, titer against* Pg*, and inflammatory markers. The multivariate associations among them were examined in Poisson regression models as follows: (1) model with periodontal disease in 2003 as a dependent variable, using cross-sectional data in 2003 and (2) model with periodontal disease at follow-up as a dependent variable, using longitudinal data. In the second model, titer against* Pg* and inflammatory markers were entered as independent variables. HsCRP, leukocytes, and BMI were treated as inflammatory markers, because obesity may be linked to chronic inflammation. As potential confounders, age, toothbrushing frequency, and smoking were included in the model because they are known to increase the risk of periodontal disease [3]. Fasting glucose, triglycerides, HDL, and blood pressure were also included in the model, because they are possible to be associated with inflammatory markers. Prevalence ratios (PRs) and 95% confidence intervals (CIs) were calculated. SPSS software (version 19.0 for Windows; IBM SPSS Japan, Tokyo, Japan) was used for data analyses.

## 3. Results

The percentage of participants having periodontal disease was 47.4% in 2003. Oral and systemic health status in 2003 and 2011 is shown in Table 1. There were gender differences in the number of the present teeth, smoking habit, toothbrushing frequency, and alcohol consumption. Serum antibody titer against* Pg* and hsCRP did not show a gender difference. Among the 2003 data, the associations of periodontal disease with titer against* Pg* and with systemic health are shown in Table 2. When the Poisson regression model included covariates with a significance level for retention of *P* < 0.2 on bivariate analysis, high titer against* Pg* was significantly associated with the periodontal disease in both males and females. In females, overweight was significantly associated with periodontal disease.

Among longitudinal data, periodontal disease persisted 8 years later in 81.1% of males with periodontal disease in 2003 and 50.0% of females with periodontal disease in 2003 had persistent periodontal disease 8 years later (Table 3). Among periodontally healthy males in 2003, 25.7% developed periodontal disease 8 years later, while, for females, 20.3% developed periodontal disease 8 years later. When we evaluated the association between hsCRP and possible related factors, such as fasting glucose, triglycerides, HDL, blood pressure, and leukocytes, these factors were significantly associated with hsCRP. Acute bacterial and viral infections (*n* = 0), myocardial infarction (*n* = 1), rheumatoid arthritis (*n* = 1), and cancer (*n* = 21) were not associated with hsCRP. Poisson regression analyses showed that levels of hsCRP were significantly related to development of periodontal disease 8 years later in periodontally healthy females in 2003 (PR 6.10; 95% CI: 1.22–30.31; *P* value 0.009), even when age, smoking, toothbrushing frequency, and possible hsCRP-related factors were included in the model (Model 1 in Table 4). Leukocytes count was not associated with periodontal disease. Persistence of periodontal disease 8 years later in females was significantly associated with antibody titer against* Pg* (PR 1.16; 95% CI: 1.04–1.30; *P* value 0.006) and hsCRP (PR 3.00; 95% CI: 1.08–8.31; *P* value 0.034). The interaction between* Pg* and hsCRP was not statistically significant. To confirm the consistency of the results, we treated antibody titer against* Pg* as a categorical variable (Model 2 in Table 4). These associations were not significant in males.

When the association between titer against* Pg* in 2003 and systemic health such as lower HDL, elevated triglycerides, and blood pressure 8 years later was examined, the association was not significant.

## 4. Discussion

This study showed gender-specific associations of periodontal status with serum titer against* Pg* and with inflammatory markers in a long-term longitudinal study. Serum titer against* Pg* and inflammatory markers showed a stronger association with periodontal status in females than in males. To the best of our knowledge, this is the first study to show such gender differences in a longitudinal study.

In this study, we found that serum titer against* Pg* was high in participants with periodontal disease at a single time point, based on cross-sectional data in 2003. This finding is consistent with several studies [9–11]. Ebersole et al. [25] noted that elevated systemic antibodies against periodontal bacteria were reflective of subgingival colonization and existed as a response to bacterial infection at disease-active sites. These results suggest that elevated antibody levels against* Pg* are associated with prior destructive periodontal disease [10].

In addition, high titer against* Pg* influenced the persistence of periodontal disease over the 8-year period in females with periodontal disease at baseline, based on longitudinal data analyses. This finding is similar to the data of Craig et al. [10], who reported that individuals with progressed periodontal disease had heightened serum antibody response to* Pg *at baseline. Anderson et al. [26] observed that the IgG antibody against* Pg* produced in response to progressing periodontal disease appeared to lack functional properties such as direct cytolysis and opsonization in nonhuman primates. They speculated that the antibody response does not protect against periodontal disease in many patients. On the other hand, antibody levels to periodontal bacteria have been reported to remain elevated over a 30-month period, despite periodontal therapy [27]. Although it is possible that antibody levels against* Pg* are retained for long periods, our results suggest that high titers against* Pg* do not provide protection against periodontal disease in females, as compared with males. Generally, antibody plays an important role in defense against pathogenic bacteria. With regard to antibody responses against* Pg*, it has been suggested that elevated antibody levels are in response to pathogens, but have little effect on infections [7]. To determine more about the exact relationship between antibody level and periodontal disease in each gender, it will be necessary to repeat the study in larger numbers of subjects and to assess* Pg* colonization.

Gender differences in the longitudinal association between antibody titer against* Pg* and periodontal disease might be explained by disparities in immune responses. Although sex differences in immune response remain incompletely understood, presumably, sex-specific genetic architecture accounts for dimorphisms in immune response and host susceptibility, exerting profound effects on multiple immunologic parameters [28]. The X-chromosome encodes approximately 1,100 genes, related to immunity [29]. Females have two X-chromosomes, which provides the added biological advantage of the cellular mosaicism that is associated with X-inactivation, whereas males are more vulnerable to X-linked diseases as they have a single X-chromosome [29]. Sex differences in immunity would be associated with cellular functions dependent on genes located on the X-chromosome [30]. We speculate that protection from periodontal disease by antibody against* Pg* differs between males and females, probably due to sexual dimorphisms in immunoinflammatory response that is influenced by sex-specific genetic architecture.

When we investigated the association between inflammatory markers and progression of periodontal disease, higher levels of hsCRP were significantly associated with development and persistence of periodontal disease in females, but not in males. While Paraskevas et al. [31] reported strong evidence by meta-analyses of cross-sectional studies that CRP in periodontal disease was elevated as compared with healthy individuals, our longitudinal study indicated gender-specific effects of CRP levels on the progression of periodontal disease. The difference in the inflammatory response in females compared to males has long been noted [32]. These differences by gender may be due to sex hormone, estrogen. However, this does not provide sufficient evidence because estrogen production seems to diminish in our female subjects with menopause. Conversely, a lot of evidences suggest that the declining function of the ovaries in females is associated with spontaneous increases in systemic proinflammatory cytokines [33, 34]. Inflammation is known to cause periodontal disease [2, 35, 36]. The present results suggest that the relationship between periodontal disease and systemic inflammation presumably differs by gender because of the differences in the inflammatory response by gender in regard to proinflammatory cytokine. The exact mechanism by which estrogen modulates proinflammatory cytokine activity has not yet been conclusively clarified [33, 37], and further analyses are needed to fully understand the details of the mechanism of the interference by estrogen.

There were several limitations to the current study. We used the same method to measure titers against* Pg* as reported by Kudo et al. [11], and the percentage of participants with ≥ 1.0 titers in our study and the study by Kudo et al. was 88% and 76%, respectively. In addition, when we analyzed receiver operating characteristics (ROC) curves for periodontal disease (presenting of PD ≥4 mm), the optimal cut-off point of titers against* Pg* was 7.75 (1.68 in Kudo et al.) and sensitivity, specificity, and the areas under the ROC curve were 0.663, 0.638, and 0.691. Hence, our participants may have had higher titers against* Pg*, as compared to those in the study by Kudo et al. Second, we did not have any information on oral health behavior such as regular dental visits and receipt of dental treatment. These would affect periodontal status, and thus our findings could reflect confounding by omitting these variables. Third, we did not measure the sign of periodontal disease such as clinical attachment loss. It has been suggested that the elevated antibody levels are in response to pathogens and are expected to be influenced by the size of the area of infection rather than by the history of tissue destruction [11]. At baseline, periodontal pocket depth would be acceptable in investigating the association between titers against* Pg* and periodontal condition. It is not beyond the realm of possibility that titers against* Pg *are related to attachment loss 8 years later. Finally, socioeconomic status was not included as a factor in this analysis. Future studies should include this, because it is possible that socioeconomic status is connected with periodontal and systemic health status.

Even considering the above limitations, the present study illustrates that females with high titer against* Pg* and high level of inflammatory marker are more likely to have periodontal disease some years later, while males have a relatively weak relationship among the titer against* Pg*, inflammatory markers, and periodontal disease.

## Figures and Tables

**Table 1:** Characteristics of study subjects in 2003 and 2011.

Variables	2003				2011			
	Males (*n* = 295)	Females (*n* = 116)	All	*P* value	Males (*n* = 295)	Females (*n* = 116)	All	*P* value
The number of the present teeth (median [25%, 75%])	27 (24, 28)	26 (21, 28)	26 (23, 28)	0.001	26 (22, 28)	24 (20, 27)	25 (22, 28)	0.003
Periodontal disease (PD ≥ 4 mm) (%)	48.5	44.8	47.4	0.505	52.5	33.6	47.2	0.001
Serum antibody titer against *Pg* ^*^	7.7 (3.3, 15.6)	8.6 (3.5, 21.8)	7.9 (3.4, 17.0)	0.363				

Age (median [25%, 75%])	57 (48, 68)	60 (50, 69)	57 (49, 68)	0.166	65 (56, 76)	68 (58, 77)	65 (57, 76)	0.158
More than 3 times daily toothbrushing	28.1	50.0	34.3	<0.001				
Current/past smoking (%)	63.7	3.4	46.7	<0.001	63.7	3.4	46.7	<0.001
Current alcohol consumption (%)^†^	76.3	37.1	65.2	<0.001	69.5	29.8	58.4	<0.001

Overweight (%)	30.2	21.6	27.7	0.079	28.8	27.6	28.5	0.804
Elevated hsCRP (≥1 mg/L) (%)^*^	18.6	17.2	18.2	0.740				
Elevated fasting glucose (≥100 mg/dL) (%)	62.2	51.7	59.4	0.048	55.9	37.9	50.9	0.001
Reduced HDL (males < 40, females < 50 mg/dL) (%)^‡^	3.7	5.2	4.1	0.508	1.4	2.6	1.7	0.386
Elevated triglycerides (≥150 mg/dL) (%)^§^	32.9	25.0	30.7	0.119	33.6	37.9	34.8	0.402
Elevated blood pressure (≥130/≥85 mmHg) (%)^‖^	34.6	25.9	32.1	0.089	29.8	16.4	26.0	0.005

Elevated leukocytes (≥9.0 × 10^9^ cells/L) (%)	4.4	0	3.2	0.024	1.4	0	1.0	0.581
Leukocytes (median [25%, 75%]) (10^9^ cells/L)	5.5 (4.7, 6.6)	5.2 (4.5, 6.0)	5.4 (4.6, 6.3)	0.003	5.1 (4.3, 6.0)	4.7 (4.1, 5.5)	5.0 (4.3, 5.8)	0.007
Neutrophils (median [25%, 75%]) (10^9^ cells/L)	3.2 (2.6, 3.9)	3.0 (2.5, 3.6)	3.1 (2.6, 3.8)	0.038	3.0 (2.4, 3.5)	2.7 (2.1, 3.3)	2.9 (2.3, 3.5)	0.013
Acidocytes (median [25%, 75%]) (10^9^ cells/L)	0.19 (0.01, 0.25)	0.12 (0.08, 0.20)	0.15 (0.09, 0.24)	0.003	0.13 (0.08, 0.23)	0.10 (0.06, 0.18)	0.13 (0.08, 0.21)	<0.001
Monocytes (median [25%, 75%]) (10^9^ cells/L)	0.31 (0.25, 0.37)	0.26 (0.22, 0.31)	0.30 (0.24, 0.36)	<0.001	0.29 (0.24, 0.36)	0.24 (0.21, 0.29)	0.28 (0.23, 0.34)	<0.001

^*^Serum antibody titer against *Pg* and hsCRP were measured in preserved serum samples in 2003.

^†^Males, *n* = 292; females, *n* = 114, in 2011.

^‡^HDL < 40 mg/dL in males and <50 mg/dL in females for reduced HDL.

^§^Triglycerides ≥ 150 mg/dL or drug treatment for dyslipidemia.

^‖^Systolic blood pressure ≥130 mmHg or diastolic blood pressure ≥85 mmHg or antihypertensive drug treatment.

PD, periodontal pocket depth; hsCRP, high-sensitive C-reactive protein; HDL, high-density lipoprotein cholesterol.

**Table 2:** Prevalence ratios for periodontal disease (presence of periodontal pocket depth ≥4 mm) in 2003 in males and females.

Covariates from data of 2003	Males			Females		
	Number of those having periodontal disease (*n* = 143)	Crude PR (95% CI)	Adjusted PR^†^ (95% CI)	Number of those having periodontal disease (*n* = 52)	Crude PR (95% CI)	Adjusted PR^†^ (95% CI)
Serum antibody titer against *Pg* (%)						
Low	53 (35.3)	1	1	15 (26.8)	1	1
High	90 (62.1)	1.8 (1.4–2.3)^***^	1.6 (1.3–2.1)^***^	37 (61.7)	2.3 (1.4–3.7)^***^	2.2 (1.3–3.5)^**^
Overweight						
No	102 (49.5)	1		34 (37.4)	1	1
Yes	41 (46.1)	0.9 (0.7–1.2)		18 (72.0)	1.9 (1.3–2.8)^***^	1.7 (1.2–2.6)^*^
hsCRP						
Normal	116 (48.3)	1		40 (41.7)	1	1
Elevated hsCRP	27 (49.1)	1.0 (0.8–1.4)		12 (60.0)	1.4 (0.9–2.2)	1.0 (0.6–1.6)
Leukocytes		1.0 (0.9–1.1)			1.0 (0.9–1.1)	

Fasting glucose						
Normal	51 (45.9)	1		23 (41.1)	1	
Elevated fasting glucose	92 (50.0)	1.1 (0.8–1.4)		29 (48.3)	1.2 (0.8–1.7)	
HDL						
Normal	139 (48.9)	1		46 (41.8)		
Reduced	4 (36.4)	0.7 (0.4–1.6)		6 (100.0)^ ‡^		
Triglycerides						
Normal	99 (50.0)	1		36 (41.4)	1	
Elevated triglycerides	44 (45.4)	0.9 (0.7–1.2)		16 (55.2)	1.3 (0.9–2.0)	
Blood pressure						
Normal	95 (49.2)	1		37 (43.0)	1	
Elevated blood pressure	48 (47.1)	1.1 (0.8–1.4)		15 (50.0)	1.2 (0.8–1.8)	

Age						
<60 yrs	70 (40.2)	1	1	24 (42.1)	1	
≥60 yrs	73 (60.3)	1.5 (1.2–1.9)^**^	1.4 (1.1–1.8)^**^	28 (47.5)	1.1 (0.8–1.7)	
Toothbrushing frequency						
<3 times/day	107 (50.5)	1		29 (50.0)	1	
≥3 times/day	36 (43.4)	0.9 (0.7–1.1)		23 (39.7)	0.8 (0.5–1.2)	
Smoking						
Never	46 (43.0)	1	1	50 (44.6)	1	
Current/Past	97 (51.6)	1.2 (0.9–1.6)	1.1 (0.9–1.5)	2 (50.0)	1.1 (0.4–3.0)	
Alcohol consumption						
No	34 (48.6)	1		34 (46.6)	1	
Current	109 (48.4)	1.0 (0.8–1.3)		18 (41.9)	0.9 (0.6–1.4)	

Poisson regression analysis with periodontal disease (periodontal pocket depth ≥4 mm) in 2003 as the dependent variable and serum antibody titer against *Pg*, overweight, hsCRP, leukocytes fasting glucose, HDL, triglycerides, blood pressure, age, smoking, and drinking in 2003 as the independent variables.

^*^ *P* < 0.05, ^**^ *P* < 0.01, and ^***^ *P* < 0.001.

^†^Adjusted prevalence ratios in the final multivariable model after including covariates with a significance level for retention of *P* < 0.2.

^‡^PR was not calculated because of complete separation (the number of females with periodontally healthy and reduced HDL was 0).

HsCRP, high-sensitive C-reactive protein; HDL, high-density lipoprotein cholesterol; PR, prevalence ratio; CI, confidence interval.

**Table 3:** Changes in periodontal disease between 2003 and 2011.

Variables from data of 2003	Periodontally healthy in 2003				Periodontal disease in 2003			
	Males		Females		Males		Females	
	In 2011		In 2011		In 2011		In 2011	
	Healthy	Periodontal disease	Healthy	Periodontal disease	Healthy	Periodontal disease	Healthy	Periodontal disease
	*n* = 113	*n* = 39	*n* = 51	*n* = 13	*n* = 27	*n* = 116	*n* = 26	*n* = 26
Serum antibody titer against *Pg* ^†‡^	5.5 (1.3, 10.9)	5.7 (2.9, 10.2)	5.2 (2.9, 11.7)	6.0 (3.6, 11.1)	11.1 (5.8, 24.5)	10.8 (4.7, 26.3)	9.9 (2.6, 22.5)	19.5 (10.9, 39.1)^*^
High serum antibody level (≥7.9) to *Pg* (%)^‡^	35.4	38.5	37.3	30.8	66.7	62.1	57.7	84.6^*^

Overweight (%)^‡^	27.4	43.6	9.8	15.4	44.4	25.0^*^	26.9	42.3

Elevated hsCRP (≥1.0) (%)^‡^	15.9	25.6	5.9	38.5^**^	14.8	19.8	7.7	38.5^**^
Leukocytes (10^9^ cells/L)^†§^	5.3 (4.5, 6.5)	5.5 (4.6, 6.6)	5.2 (4.6, 5.5)	5.0 (4.6, 6.5)	5.3 (4.8, 6.5)	5.8 (4.8, 6.7)	5.2 (4.3, 6.3)	5.2 (4.4, 6.1)

^*^ *P* < 0.05, ^**^ *P* < 0.01.

^†^Median (25%, 75%).

^‡^Comparison between participants with or without periodontal disease in 2011 by bivariate analysis.

^§^Continuous variable of leukocytes was used, because of small number of elevated leukocytes (≥9.0 × 10^9^ cells/L).

HsCRP, high-sensitive C-reactive protein.

**Table 4:** Prevalence ratios for periodontal disease in 2011.

	Periodontally healthy in 2003		Periodontal disease in 2003	
	Males	Females	Males	Females
	PR (95% CI)^†^	PR (95% CI)^†^	PR (95% CI)^†^	PR (95% CI)^†^
*Model 1 *				
Serum antibody titer against *Pg *	1.04 (0.94–1.14)	1.11 (0.93–1.32)	1.00 (0.97–1.03)	1.16 (1.04–1.30)^**^
Overweight				
No	1	1	1	1
Yes	1.01 (0.58–1.75)	0.93 (0.39–2.24)	0.82 (0.66–1.01)	0.72 (0.28–1.87)
hsCRP				
Normal	1	1	1	1
Elevated hsCRP	1.27 (0.70–2.29)	6.10 (1.22–30.31)^**^	0.99 (0.82–1.19)	3.00 (1.08–8.31)^*^
Leukocytes^‡^	0.96 (0.86–1.05)	0.90 (0.67–1.20)	1.01 (0.98–1.04)	0.97 (0.87–1.10)

*Model 2 *				
Serum antibody titer against *Pg *				
Low	1	1	1	1
High	1.05 (0.61–1.82)	0.85 (0.25–2.94)	0.96 (0.81–1.13)	2.21 (1.04–4.73)^*^
Overweight				
No	1	1	1	1
Yes	1.03 (0.59–1.80)	1.04 (0.31–3.45)	0.81 (0.65–1.00)	0.80 (0.23–2.74)
hsCRP				
Normal	1	1	1	1
Elevated hsCRP	1.25 (0.70–2.26)	5.96 (1.31–27.13)^**^	1.00 (0.83–1.20)	3.70 (1.07–12.80)^*^
Leukocytes^‡^	0.96 (0.86–1.05)	0.90 (0.66–1.22)	1.01 (0.98–1.04)	0.99 (0.88–1.12)

Dependent variable, 1: periodontal disease in 2011, 0: periodontally healthy in 2011.

Model 1: continuous variable of serum antibody titer against *Pg* was included.

Model 2: categorical variable of serum antibody titer against *Pg* was included.

^*^ *P* < 0.05, ^**^ *P* < 0.01.

^†^Adjusted by age, smoking, toothbrushing frequency, triglycerides, HDL, fasting glucose, and systolic blood pressure in 2003.

^‡^Continuous variable of leukocytes was used, because of small number of elevated leukocytes.

HsCRP, high-sensitive C-reactive protein; PR, prevalence ratio; CI, confidence interval.
